# Incidence of dementia in a Kashmiri migrant population

**DOI:** 10.4103/0972-2327.56313

**Published:** 2009

**Authors:** Sunil Kumar Raina, Kamal Kishore Pandita, Sushil Razdan

**Affiliations:** Departments of Community Medicine and Medicine, Acharya Shri Chander College of Medical Sciences, Sidhra, Jammu (J & K), India

**Keywords:** Epilepsy, social responsibility, treatable

## Abstract

**Background:**

Mishriwala is one of five exclusive clusters of Kashmiri migrants established in 1990 to accommodate Kashmiri Pandit families who left Kashmir valley in the wake of militancy. Mishriwala migrant camp has seen minimal immigration and out-migration since its establishment. In an earlier study we reported on the prevalence of dementia amongst a Kashmiri migrant population. That study was conducted in the migrant camp at Mishriwala, 12 km west of Jammu city, the winter capital of Jammu and Kashmir State. We have developed standardized study methods and instruments for use in the Kashmiri-speaking population, which we used for screening for dementia during the prevalence study. We now report the results of a 1-year prospective study carried out to find out the incidence of dementia in the same population.

**Aim:**

To ascertain the incidence of dementiain the Kashmiri Pandit population aged 60 years and above.

**Materials and Methods:**

A 1-year, prospective, epidemiological study of 186 subjects aged 60 years and above, using cognitive and functional ability screening and clinical evaluation.

**Results:**

The incidence of dementia in this population was 5.34 cases per 1000 person-years.

## Introduction

Alzheimer's disease and other dementias are already a major public health problem among the elderly in industrialized countries. These dementias could have a devastating impact on developing countries, where the proportion of the aged in the population is increasing very rapidly. By the year 2020, approximately 70% of the world's population aged 60 and above will be living in developing countries, and 14–20% of them will be in India.[1] This ageing population will place a great burden on the health care systems in developing countries. The World Health Organization (WHO) estimates that by the year 2020, Africa, Asia, and Latin America will have more than 55 million people with senile dementia.[1]

The existing disease burden in communities can be estimated by cross-sectional prevalence studies but the rate at which new disease develops can be measured only with prospective incidence studies. There are several published reports on the prevalence of dementia in developing countries in Asia and Africa,[2–7] but few reports on the incidence.[8–11] We have previously reported the prevalence of dementia in Mishriwala migrant camp, a community of Kashmiri migrants 12 km west of Jammu city in northern India.[12] In this article, we report the results of a 1-year incidence study of dementia in the same population.

## Materials and Methods

### Study population

The study population for our earlier prevalence study[12] consisted of all 200 individuals aged 60 years and above from Mishriwala migrant camp. We used the age given in the ration card to identify eligible subjects. Subsequently, their ages were confirmed in person by reference to personal and historical sentinel events, as is standard practice in research in developing countries.[13] At study entry, 13 subjects already had dementia. Two individuals were lost during the follow-up, one of whom was a case of dementia. Thus, after excluding these two cases and the 12 remaining cases of dementia, we had a cohort comprising 186 individuals above 60 years of age and without evidence of dementia.

### Instrument development

The first stage of the study was devoted to the development of an instrument suited to the study population. The instrument had to be culturally and linguistically acceptable to the local Kashmiri-speaking population. We developed this instrument by means of a systemic, iterative process. A team composed of a neurologist, a physician trained in neurology, and an epidemiologist, all Kashmiri's, selected items from the English version of the Mini Mental Status Examination (MMSE) and translated them into Kashmiri. The Kashmiri version was back-translated into English by a group of doctors (also all Kashmiri's), independent of the first group. The selected items were first tested on a group of 30 individuals selected randomly from among 100 bilingual members of our cohort. We ensured that the results obtained with the English and Kashmiri versions of the MMSE were comparable by administering both versions to the same 30 individuals and evaluating the scores.

Using the same interactive process, we developed an instrument in the questionnaire form for assessment of functional ability: the Everyday Abilities Scale for India (EASI). This was administered to the subject and a family member. It comprised questions related to the routine activities of an elderly adult in a rural Indian setting. The test items were examined and modified to maximize ease of administration and comprehension; we also made sure that the questions were acceptable to the study participants and that the administration and scoring were reliable. This helped us to obtain the functional ability data in subjects who were cognitively untestable because of sensory impairment, illness, or severe dementia.

### Screening

The MMSE has been used in studies as a screening tool to detect dementia.[14,15] In this study, the Kashmiri version of the MMSE was administered by trained interviewers to the 186 individuals of the cohort at their residences. The MMSE score was used to establish the presence or absence of a dementia syndrome and the stage of severity. Any subject with an MMSE score below 24 (out of a maximum possible score of 30) was evaluated in detail clinically.

Clinical evaluation was carried out by a neurologist with the help of a physician trained in neurology. The evaluation included detailed history, general physical examination, neurological examination, and the examination of the mental status of the subject. Patients diagnosed with dementia had further investigations done, e.g., hemogram, serum biochemistry, thyroid function test, and MRI of brain.

## Results

Of the 186 individuals in our study cohort, 47% were males and 53% were females. Fifty percent of the individuals were illiterate. The number of patients in the different age-groups is shown in Table 1. The demographic characteristics and the incidence of dementia in the study population are shown in Table 2.

**Table 1:** Age and gender distribution of the study population at Mishriwala

Age (Years)	Male	Female
60-64	30 (33)	47 (48)
65-69	29 (32)	31 (31)
70-74	18 (20)	16 (16)
≥ 75	12 (13)	03 (3)
Total	89 (47)	97 (53)

Figures in parenthesis are percentages.

**Table 2:** Demographic characteristics and incidence of dementia in the study population (N = 186)

	Total number of subjects	Number of cases of dementia	Incidence of dementia (per 1000/year)
Age-group			
60-64	77	0	
65-69	60	0	
70-74	34	0	
≥ 75	15	1	5.34
Gender			
Male	90	0	
Female	96	1	10.20
Literacy			
Illiterate	93	0	
Primary	29	1	34.48
Education			
Matriculation	42	0	
Above			
matriculation	22	0	

The incidence was calculated by combining the units of population and time into a single measure of person-time which, in our case, was person-years. Over the 1-year follow-up period one participant aged ≥ 75 years developed features suggestive of dementia. This gave an incidence rate for dementia of 5.34 per 1000 person-years.

The hemogram and the renal and liver function tests in the patient with dementia was normal. MRI of the brain was unremarkable.

## Discussion

The incidence of a disease is the number of new cases of the disease that comes into being during a specified period of time. We have previously reported the prevalence rates for dementia in Kashmiri migrants at Mishriwala migrant camp in Jammu. We now report the results of a 1-year incidence survey of dementia in the same community.

The incidence rate as defined in our report is a measure of events occurring during the observation period, an indicator of a hazard. It allows hypothesis to be generated regarding the differential distribution of risk and the protective factors. Suggestions that the incidence of Alzheimer's disease is lower in Asia than in Europe and North America[16] have in fact been based on the results of only a few Asian incidence studies of Alzheimer's disease–one from Japan,[9] one from China,[8] and one from Taiwan[10]; these studies reported rates ranging from 5.1 to 8.9 per 1000 person-years among those aged ≥ 65. The overall dementia incidence rate among those aged 60 and above at Mishriwala migrant camp was 5.34 per 1000 person-years, slightly higher than that reported from the Ballabgarh study.[17]

As discussed in our previous paper on the prevalence of dementia in Mishriwala, a major advantage in our study was that we had a stable ‘captive’ population that, although small in size, was linguistically and culturally uniform.

Dementia appears to be very rare in the native Kashmiri population.[18] Responsible for dementia in study population.

Given the short duration of follow-up in our study and the small sample size, generalization of our results may not be possible. Further research, with a large sample taken from all migrant clusters or with multiple samples from different parts of Jammu and Kashmir State, might help us identify the protective factors and provide testable hypothesis for further investigations.
